# Datasets, processing and refinement details for *Mtb*-AnPRT: inhibitor structures with various space groups

**DOI:** 10.1016/j.dib.2017.10.051

**Published:** 2017-10-31

**Authors:** Genevieve L. Evans, Daniel P. Furkert, Nacim Abermil, Preeti Kundu, Katrina M. de Lange, Emily J. Parker, Margaret A. Brimble, Edward N. Baker, J. Shaun Lott

**Affiliations:** a Maurice Wilkins Centre for Molecular Biodiscovery and School of Biological Sciences, University of Auckland, 3 Symonds Street, Auckland 1142, New Zealand; b Maurice Wilkins Centre for Molecular Biodiscovery and School of Chemical Sciences, University of Auckland, 23 Symonds Street, Auckland 1142, New Zealand; c Maurice Wilkins Centre for Molecular Biodiscovery, Biomolecular Interaction Centre and Department of Chemistry, University of Canterbury, P.O. Box 4800, Christchurch 8140, New Zealand

**Keywords:** Crystallography, Macromolecules, X-ray diffraction, Ligand binding, Structure-based inhibitor design

## Abstract

There are twenty-five published structures *of Mycobacterium tuberculosis* anthranilate phosphoribosyltransferase (*Mtb*-AnPRT) that use the same crystallization protocol. The structures include protein complexed with natural and alternative substrates, protein:inhibitor complexes, and variants with mutations of substrate-binding residues. Amongst these are varying space groups (*i.e. P*2_1_, *C*2, *P*2_1_2_1_2, *P*2_1_2_1_2_1_). This article outlines experimental details for 3 additional *Mtb*-AnPRT:inhibitor structures. For one protein:inhibitor complex, two datasets are presented – one generated by crystallization of protein in the presence of the inhibitor and another where a protein crystal was soaked with the inhibitor. Automatic and manual processing of these datasets indicated the same space group for both datasets and thus indicate that the space group differences between structures of *Mtb*-AnPRT:ligand complexes are not related to the method used to introduce the ligand.

**Specifications Table**

**Table:** 

Subject area	*Proteomics and Biochemistry*
More specific subject area	*Structural biology*
Type of data	*Tables, figures and X-ray diffraction images*
How data was acquired	*X-ray macromolecular crystallography;*
	*Australian Synchrotron MX1 and MX2 beamlines*
Data format	*Unprocessed, processed, deposited with crystal packing analyzed.*
Experimental factors	*Protein crystals soaked and co-crystallized with ligands*
Experimental features	*Contrast of the different protein crystal packing associated with different inhibitors and/or introduced by soaking or co-crystallization.*
	*X-ray diffraction datasets for co-crystallization and soaked-in experiments with the same inhibitor indicate space group changes are independent of method used to introduce the inhibitor.*
Data source location	*Data collected in Melbourne, Australia.*
Data accessibility	*The atomic coordinates and observed structure factors are available from Protein Data Bank with accession numbers:*5BO2*,*5BO3*and*5BNE.
	*The X-ray diffraction image files corresponding to datasets from two experiments are hosted by Mendeley:*
	http://dx.doi.org/10.17632/2zrfgv34nb.1
	http://dx.doi.org/10.17632/xgn5z8jnr7.1
Related research article	*Anthranilate phosphoribosyltransferase: Binding determinants for 5′-phospho-alpha-d-ribosyl-1′-pyrophosphate (PRPP) and the implications for inhibitor design “in press”.*
	https://doi.org/10.1016/j.bbapap.2017.08.018

**Value of the data**

• *Mtb*-AnPRT is a target of interest in developing novel anti-tuberculosis agents. This protein's capacity to crystallize thus yield new protein:ligand complexes makes it of interest for structure-based inhibitor design.
• Previously *Mtb*-AnPRT:ligand complexes have been found with different space groups (*e.g. P*2_1_, *C*2, *P*2_1_2_1_2, and *P*2_1_2_1_2_1_), generated by using the same crystallization protocol.
• *Mtb*-AnPRT:inhibitor complex structures described herein have *C*2 and *P*2_1_ space groups. These structures were solved using X-ray diffraction datasets from protein:ligand crystals generated by streak-seeded with wild-type *P*2_1_2_1_2_1_ crystal.
• For one inhibitor, X-ray diffraction datasets are presented for both co-crystallization and soaked crystal experiments. Space group *C*2 occurred in both datasets and indicates the space group change between ligand-free and inhibitor-bound structures are independent of method used to introduce the inhibitor.
• X-ray diffraction datasets utilizing different methods of ligand introduction and yielding equivalent protein:ligand structures are typically not made available. These publically available datasets in the context of multiple space groups observed for *Mtb*-AnPRT:ligand structures, and the analysis presented herein, demonstrate that space group changes can be independent of co-crystallization and soaking methods of ligand introduction. This has relevance to academic and industrial researchers who are pursuing structure-based inhibitor design.

## 1 Data

### 1.1 Overview

The experimental and data processing details for 3 new protein:ligand structures of *Mycobacterium tuberculosis* anthranilate phosphoribosyl transferase (*Mtb*-AnPRT) are described herein (Table 1). The protein structures are complexed with *Mtb*-AnPRT inhibitors characterized in [1] and annotated as:

• **8k** (2-(2-carboxyphenylamino)-5-(5-phosphonopentyloxy)benzoic acid)
• **8j** (2-(2-carboxyphenylamino)-5-(4-phosphonobutoxy)benzoic acid)
• **8i** (2-(2-carboxyphenylamino)-5-(3-phosphonopropoxy)benzoic acid)

**Table 1:** Summary of *Mtb*-AnPRT structures described herein.

PDB ID	Ligand ID	Solvent content	Unit Cell				Space group	Resolution (Å)	Chains	PDB DOI
			β (°)	A (Å)	B (Å)	C (Å)				
5BO2	**8i**	46%	111°	94	78	103	*C*2	2.00	A,B	10.2210/pdb5bo2/pdb
5BO3	**8j**	46%	111°	95	78	103	*C*2	1.75	A,B	10.2210/pdb5bo3/pdb
5BNE	**8k**	46%	91°	77	78	117	*P*2_1_	2.15	A,B,C D	10.2210/pdb5bne/pdb

These structures were determined with protein crystallized in presence of imidazole-malate and PEG4000. Crystallization drops seeded with crystal nuclei from a pre-existing *Mtb*-AnPRT crystal generate better crystal morphology for *Mtb*-AnPRT [2]. All new structures presented herein were generated by streak-seeding using wild-type *P*2_1_2_1_2_1_ crystals. Also presented are two X-ray diffraction datasets corresponding to protein crystals either soaked and co-crystallized with the same inhibitor (Table 2).

**Table 2:** Crystallization of complexes, along with space group and unit cell from data processing.

PDB ID	Ligand(s) bound	[Protein] mg mL^−^^1^	Reservoir condition	Cryoprotectant/Soak	Notes	Space group	Unit cell
5BO2	**8i :**	3.0	0.2 M imidazole.malate, pH 7.0, 9% PEG4000	0.2 M imidazole.malate, pH 7.0, 15% PEG4000, 1 mM **8i**	Streak seeded; **soaked crystal** for 4 h;	*C*2	94×78×103 Å
					2 day old crystal		
					**X-ray images available at:**http://dx.doi.org/10.17632/xgn5z8jnr7.1		*ß*β=111°
Not applicable	**8i :**	3.0	0.2 M imidazole.malate, pH 7.0, 11% PEG4000, 1 mM **8i**	0.2 M imidazole.malate, pH 7.0, 15% PEG4000, 1 mM **8i**	Streak seeded; **protein co-crystallized with ligand**;	*C*2	95×78×103 Å
					2 day old crystal		
							β=111°
					**X-ray images available at:**http://dx.doi.org/10.17632/2zrfgv34nb.1		
5BO3	**8j :**	3.0	0.2 M imidazole.malate, pH 7.0, 11% PEG4000,	0.2 M imidazole.malate, pH 7.0, 15% PEG4000, 1 mM **8j**	Streak seeded; **soaked crystal** for 10 min;	*C*2	95×78×103 Å

					2 day old crystal		
							β=111°
5BNE	**8k :**	3.1	0.2 M imidazole.malate, pH 7.0, 15% PEG4000, 1 mM **8k**	No cryo used, because crystallization condition contained 15% PEG4000	Streak seeded; **protein co-crystallized with ligand**;	*P*2_1_	77×78×117 Å

					2 day old crystal		
							β=91°

Of the previously published structures of *Mtb*-AnPRT, 25 were determined from protein crystallized using this protocol (Table 3). Most of these structures have a ligand bound (*e.g.* inhibitor or substrate) and/or are protein variants with mutations in substrate-binding residues (Table 3). Amongst both cohorts (Table 1, Table 3) several different space groups have been observed, including *P*2_1_, *C*2, *P*2_1_2_1_2, *P*2_1_2_1_2_1_. Amongst the 28 structures referred to in this article (Table 1, Table 3), similar unit cells correspond to each space group.

**Table 3:** Space groups, unit cells and other information for previously published *Mtb*-AnPRT structures crystallized in imidazole-malate and PEG4000.

PDB ID	Ref	Solvent content	Unit Cell				Space Group	Resolution (Å)	Chains	Notes	PDB DOI
			β (°)	A (Å)	B (Å)	C (Å)					

3QS8	[2]	46%	90°	78	81	111	*P*2_1_	2.00	A,B,C,D	Co-crystal	10.2210/pdb3qs8/pdb
3UU1	[2]	45%	90°	78	111	80	*P*2_1_	1.82	A,B,C,D	Co-crystal	10.2210/pdb3uu1/pdb
3R6C	[2]	44%	110°	94	78	100	*C*2	1.83	A,B	Co-crystal	10.2210/pdb3r6c/pdb
4IJ1	[5]	45%	111	95	78	102	*C*2	1.79	A,B	Co-crystal	10.2210/pdb4ij1/pdb
4X58	[6]	50%	111	95	78	101	*C*2	1.75	A,B	Mutant	10.2210/pdb4x58/pdb
4X59	[6]	50%	112	95	78	102	*C*2	1.80	A,B	Mutant	10.2210/pdb4x59/pdb
4X5A	[6]	49%	112	94	78	102	*C*2	1.93	A,B	Mutant	10.2210/pdb4x5a/pdb
4X5B	[6]	45%	111	94	78	100	*C*2	2.47	A,B	Mutant	10.2210/pdb4x5b/pdb
4X5C	[6]	49%	111	94	78	101	*C*2	2.33	A,B	Mutant	10.2210/pdb4x5c/pdb
4X5E	[6]	50%	110	95	79	101	*C*2	1.77	A,B	Mutant	10.2210/pdb4x5e/pdb
4GIU	[5]	46%	90	111	81	79	*P*2_1_2_1_2	1.67	A,B	Co-crystal	10.2210/pdb4giu/pdb
4GKM	[5]	46%	90	111	81	78	*P*2_1_2_1_2	1.67	A,B	Co-crystal	10.2210/pdb4gkm/pdb
3QR9	[2]	57%	90	79	92	120	*P*2_1_2_1_2_1_	1.87	A,B	Ligand-free	10.2210/pdb3qr9/pdb
4M0R	[5]	56%	90	79	92	120	*P*2_1_2_1_2_1_	1.96	A,B	Co-crystal	10.2210/pdb4m0r/pdb
4N5V	[7]	57%	90	80	93	121	*P*2_1_2_1_2_1_	1.90	A,B	Soak	10.2210/pdb4n5v/pdb
4N8Q	[7]	56%	90	80	91	120	*P*2_1_2_1_2_1_	2.08	A,B	Soak	10.2210/pdb4n8q/pdb
4N93	[7]	57%	90	80	92	121	*P*2_1_2_1_2_1_	2.03	A,B	Soak	10.2210/pdb4n93/pdb
4OWM	[7]	60%	90	79	92	121	*P*2_1_2_1_2_1_	1.99	A,B	Soak	10.2210/pdb4owm/pdb
4OWN	[7]	60%	90	80	92	121	*P*2_1_2_1_2_1_	2.11	A,B	Soak	10.2210/pdb4own/pdb
4OWO	[7]	60%	90	79	92	121	*P*2_1_2_1_2_1_	1.99	A,B	Soak	10.2210/pdb4owo/pdb
4OWQ	[7]	61%	90	79	92	122	*P*2_1_2_1_2_1_	1.89	A,B	Soak	10.2210/pdb4owq/pdb
4OWS	[7]	60%	90	80	92	121	*P*2_1_2_1_2_1_	2.43	A,B	Soak	10.2210/pdb4ows/pdb
4OWU	[7]	60%	90	79	92	121	*P*2_1_2_1_2_1_	1.89	A,B	Soak	10.2210/pdb4owu/pdb
4OWV	[7]	60%	90	80	92	120	*P*2_1_2_1_2_1_	1.90	A,B	Soak	10.2210/pdb4owv/pdb
4X5D	[6]	60%	90	80	92	121	*P*2_1_2_1_2_1_	2.30	A,B	Mutant	10.2210/pdb4x5d/pdb

*P*2_1_2_1_2_1_ is the most common space group for macromolecular structures, and it has been proposed that this is due to its capacity to accommodate repositioning, *i.e.* rotations or translations, within the asymmetric unit, without loss of crystal contacts [3]. The structure of wild-type *Mtb*-AnPRT without ligands (PDB ID: 3QR9) has previously been solved from protein crystallized in imidazole-malate and PEG4000 in the space group *P*2_1_2_1_2_1_, with two monomers (A, B) in the asymmetric unit, a unit cell of 79×92×120 Å, and 57% solvent content [2].

*Mtb*-AnPRT is a homodimeric protein with an extended “S”-shape, with each subunit containing two domains [4]. In the ligand-free structure, a single dimer (the biological assembly) is found in the asymmetric unit [2]. The two subunits of the dimer are related by a non-crystallographic two-fold symmetry axis (NCS).

### 1.2 Data for protein complexes with inhibitors

The three protein:inhibitor complexes (PDB IDs: 5BO2, 5BO3, 5BNE) were solved in the absence of metals and substrate. For these structures, the solvent content has decreased by ~ 10%, the unit cell has changed (*i.e.* dimension(s) decreased by 10–20 Å), and the space group has changed to *P*2_1_ or *C*2, compared to the ligand-free wild-type structure.

In structures of AnPRT from other prokaryote species, domain movement is observed within subunits due to substrate binding and results in compression of the homodimer by 10 Å (*e.g. D*_max_ (maximum distance) changes from 110 to 100 Å [8]). However, superposition of the subunits in the new *Mtb*-AnPRT structures onto the subunits of the ligand-free structure indicates there are no large changes (Table 4; [9]). Additionally, the longest dimension of the *Mtb*-AnPRT dimer is relatively unchanged between the ligand-free protein structure and the protein:ligand complex structures (Table 4; [10]). Thus, the changes in space group are not driven by domain movements within each subunit.

**Table 4:** Comparison *Mtb*-AnPRT subunits found in the 3 new structures to ligand-free structure.

	RMSD^a^ to chain A-3QR9 (Å)	RMSD^a^ to chain B-3QR9 (Å)	Longest dimension of dimer (Å)
3qr9.pdb:A	–	0.89	109
3qr9.pdb:B	0.89	–	
5bo2.pdb:A	0.67	0.68	110
5bo2.pdb:B	0.66	0.73	
5bo3.pdb:A	0.63	0.53	110
5bo3.pdb:B	0.62	0.73	
5bne.pdb:A	0.57	0.53	109
5bne.pdb:B	0.46	0.74	
5bne.pdb:C	0.46	0.92	109
5bne.pdb:D	0.55	0.60	

a Root mean standard difference (RMSD) between the C_alpha_ atoms

**Fig. 1:**
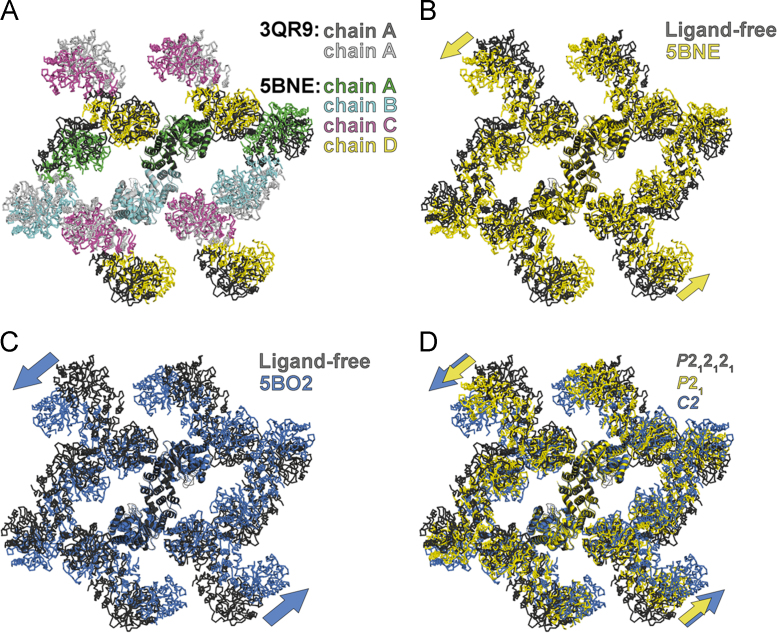
Understanding changes in crystal packing in the *Mtb*-AnPRT structures with inhibitors. (A) Superposition of the dimer (cartoon) from a *Mtb*-AnPRT:inhibitor structure defined by space group *P*2_1_, PDB ID: 5BNE (chain A, B, C and D in green, cyan, pink and yellow, respectively), onto that of the ligand-free *Mtb*-AnPRT structure (PDB ID: 3QR9 [2]; *P*2_1_2_1_2_1_; chain A and B in dark and light grey, respectively). The figure includes adjacent dimers (ribbons) in equivalent crystal layers (c-b plane in 3QR9 [2] and c-a plane in 5BNE). In (B) the superposition is re-colored with the *Mtb*-AnPRT:inhibitor structure in yellow and the ligand-free *Mtb*-AnPRT structure in dark grey. Arrows highlight the reorientation of dimers relative to each other. (C) Superposition of the dimer (cartoon) from a *Mtb*-AnPRT:inhibitor structure defined by space group *C*2, PDB ID: 5BO2 (marine blue) onto that of the ligand-free *Mtb*-AnPRT structure (PDB ID: 3QR9 [2]; *P*2_1_2_1_2_1_; dark grey). In (D) superpositions in panels B-C are combined.

The *P*2_1_ structure (PDB ID: 5BNE) contains an inhibitor annotated as **8k**, and is the third structure of *Mtb*-AnPRT with this space group determined for protein crystallized in the imidazole-malate condition. The increased components in this structure's asymmetric unit (chains A-D, vs. chains A and B; Fig. 1**A**) means that the lower symmetry described by *P*2_1_ can generate equivalent protein content in a similarly-sized unit cell as is observed with structures defined by space groups *P*2_1_2_1_2_1_ or *P*2_1_2_1_2. The ß angle of 91° could be taken to suggest that the space group should be orthorhombic (*e.g. P*2_1_2_1_2 or *P*2_1_2_1_2_1_). Both POINTLESS [11] and ZANUDA [12] indicated that *P*2_1_ was the correct space group for this dataset, however.

(A) Superposition of the dimer (cartoon) from a *Mtb*-AnPRT:inhibitor structure defined by space group *P*2_1_, PDB ID: 5BNE (chain A, B, C and D in green, cyan, pink and yellow, respectively), onto that of the ligand-free *Mtb*-AnPRT structure (PDB ID: 3QR9 [2]; *P*2_1_2_1_2_1_; chain A and B in dark and light grey, respectively). The figure includes adjacent dimers (ribbons) in equivalent crystal layers (c-b plane in 3QR9 [2] and c-a plane in 5BNE). In (B) the superposition is re-colored with the *Mtb*-AnPRT:inhibitor structure in yellow and the ligand-free *Mtb*-AnPRT structure in dark grey. Arrows highlight the reorientation of dimers relative to each other. (C) Superposition of the dimer (cartoon) from a *Mtb*-AnPRT:inhibitor structure defined by space group *C*2, PDB ID: 5BO2 (marine blue) onto that of the ligand-free *Mtb*-AnPRT structure (PDB ID: 3QR9 [2]; *P*2_1_2_1_2_1_; dark grey). In (D) superpositions in panels B-C are combined.

Structural superposition of the dimers from *Mtb*-AnPRT:inhibitor structures (PDB ID: 5BNE and 5BO2) onto the dimer present in the *P*2_1_2_1_2_1_ ligand-free *Mtb*-AnPRT structure (PDB ID: 3QR9 [2]) revealed a reorientation of the protein dimers relative to each other (Fig. 1**B and C**). The combination of these superpositions (Fig. 1**D**) indicates that the lattice in the *P*2_1_ structure corresponds to an intermediate position between the lattice observed for *P*2_1_2_1_2_1_ and *C*2 structures. We propose the subunits that are related by crystallographic symmetry elements in the *P*2_1_2_1_2_1_ structures are related by pseudosymmetry elements in the *P*2_1_ structure. Pseudosymmetry occurs where a non-crystallographic symmetry element within the asymmetric unit is close to a crystallographic symmetry operators [13]. Thus, the *P*2_1_ space group has been correctly assigned, even though the unit cell has a β of approximately 90 °.

**Fig. 2:**
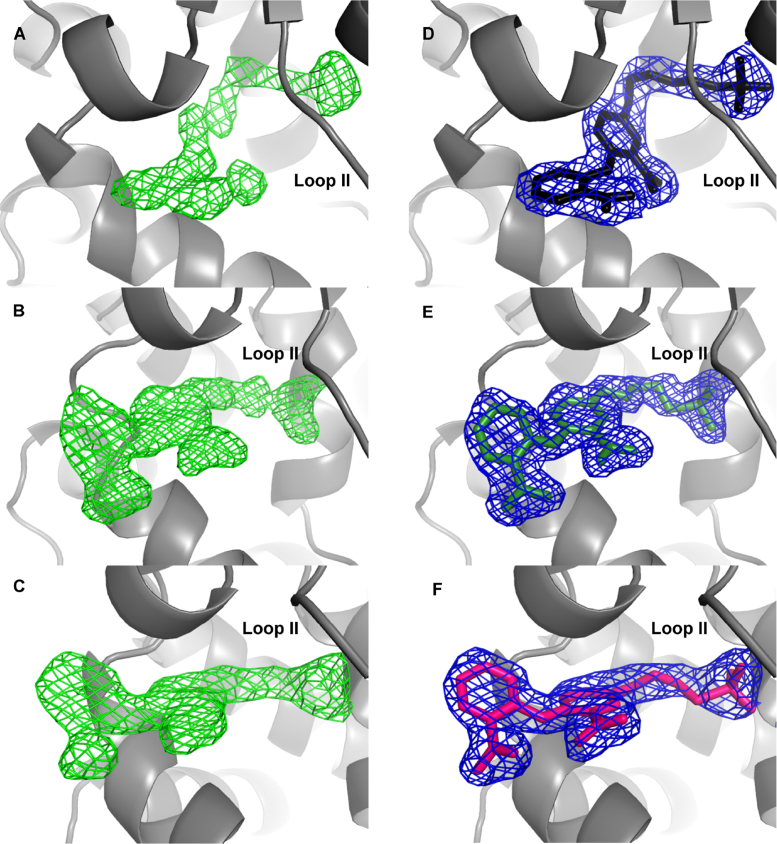
Omit and fitted map for inhibitors bound in *Mtb*-AnPRT structures. The *F*_o_-*F*_c_ map calculated (green, contoured at 3 σ) prior to the addition of ligands to the model for A) 8k, B) 8j and C) 8i (PDB entries 5BNE, 5BO3 and 5BO2, respectively). The 2*F*_o_-*F*_c_ map calculated (blue, contoured at 1 σ) after final refinement, with model including the ligands for D) 8k (black), E) 8j (green) and F) 8i (pink).

The generation of the protein: inhibitor structures, involved experiments, with all three inhibitors, using both co-crystallization and soaking-in methods. The structures deposited on the PDB correspond to those where the clearest density was observed for the inhibitor (*i.e.* modelled with full occupancy; Fig. 2). In Table 5 there are data statistics corresponding to *Mtb*-AnPRT co-crystallized with inhibitor **8i**. In Table 6 are data statistics corresponding to inhibitor **8i** soaked into a wild-type ligand-free *Mtb*-APRT crystal. In both cases, with no manual intervention, the datasets processed in XDS [14] and AIMLESS [11] as space group *C*2. Thus, in this study, there no correlation was found between space group type and the method by which the ligand was introduced (*i.e.* soaking vs. co-crystallization).

**Table 5:** Statistics for dataset of *Mtb*-AnPRT co-crystallized with inhibitor 8i.

**Data collection**	

AnPRT complexed with	**8i**
Space group	*C*2
Cell dimensions	
a, b, c (Å)	94.6, 78.1, 102.8
β (deg)	110.9
Unique reflections^a^	387946 (24179)
Resolution range (Å)^a^	48-1.95 (2.00-1.95)
*R*_merge_^a^	0.138 (1.237)
*R*_p.i.m._^a^	0.054 (0.488)
Mean *I/σ*(I)^a^	9.9 (1.5)
CC_1/2_^a^	0.997 (0.679)
Completeness (%)^a^	99.6 (94.0)
Redundancy^a^	7.6 (7.1)
Wilson B factor	20.5

^b^ The average atomic temperature factor. ^c^*R*_work_=(|*F*_obs_| - |*F*_calc_|)/|*F*_obs_| and *R*_free_=∑T (|*F*_obs_| - |*F*_calc_|)/∑T |F_obs_|, where T is a test dataset of 5% of the total reflections randomly chosen and set aside before refinement. ^d^ RMSD from ideal geometry values from Engh and Huber (1991)[15].

a Outer resolution shell is shown in parentheses.

**Table 6:** Data and refinement statistics for AnPRT complexes with inhibitors.

**Data collection**			

AnPRT complexed with	**8i**	**8j**	**8k**
PDB code	5BO2	5BO3	5BNE
Space group	*C*2	*C*2	*P*2_1_
Cell dimensions			
a, b, c (Å)	94.5, 78.0, 102.9	95.0, 78.1, 102.6	77.3, 78.4, 117.2
β (deg)	111.0	111.1	90.7
Unique reflections^a^	47170 (3415)	68993 (3669)	75079 (4387)
Resolution range (Å)^a^	47-2.00 (2.05-2.00)	48-1.75 (1.78-1.75)	47-2.15 (2.19-2.15)
*R*_merge_^a^	0.121 (0.856)	0.121 (1.762)	0.111 (0.711)
*R*_p.i.m._^a^	0.076 (0.564)	0.046 (0.688)	0.073 (0.477)
Mean *I/σ*(I)^a^	8.5 (1.5)	13.3 (1.2)	6.9 (1.5)
CC_1/2_^a^	0.993 (0.564)	0.998 (0.465)	0.993 (0.502)
Completeness (%)^a^	99.8 (97.7)	97.8 (93.7)	98.4 (77.3)
Redundancy^a^	3.4 (3.3)	7.8 (7.4)	3.0 (2.9)
Wilson B factor	15.1	16.3	21.4
**Refinement**			
Atoms, B factor (Å^2^)^b^			
Protein	4788, 25.8	4746, 24.6	9280, 29.0
Solvent	352, 29.7	402, 29.2	353, 29.5
Ligands	79, 33.2	66, 26.6	107, 30.6
*R*_work_/*R*_free_ (%/%)^a^^*,*^^c^	0.192/0.232	0.206/0.240	0.207/0.235
	(0.276/0.324)	(0.319/0.336)	(0.284/0.332)
Ramachandran outliers (%)	0.31	0.16	0.31
R.m.s.d. of			
Bond lengths (Å)^d^	0.003	0.005	0.003
Bond angles (°)^d^	0.774	0.899	0.745

a Outer resolution shell is shown in parentheses.

b The average atomic temperature factor.

c *R*_work_=(|*F*_obs_| - |*F*_calc_|)/|*F*_obs_| and *R*_free_=∑T (|*F*_obs_| - |*F*_calc_|)/∑T |F_obs_|, where T is a test dataset of 5% of the total reflections randomly chosen and set aside before refinement.

d RMSD from ideal geometry values from Engh and Huber[15].

## 2 Experimental design, materials and methods

### 2.1 Materials

Unless otherwise stated, all chemicals were obtained from Sigma-Aldrich, Scharlau, or Pure Science. The purification of *Mtb*-AnPRT, as well as the synthesis and biochemical characterization of its inhibitors annotated as **8i**, **8j** and **8k** (Table 2), are outlined in the article entitled “*Anthranilate phosphoribosyltransferase: Binding determinants for 5′-phospho-alpha-d-ribosyl-1′-pyrophosphate (PRPP) and the implications for inhibitor design”* [1].

### 2.2 Crystallization

Crystals of *Mtb*-AnPRT were obtained in hanging drops of 1–2 μL protein solution (3.0–3.1 mg mL^−1^ in final storage buffer) with the equivalent amount of reservoir solution in a fine screen of 0.2 M imidazole-malate pH 7.0–8.5, 5–15% polyethylene glycol (PEG) 4000, as previously reported [2]. Complexes were prepared either by co-crystallization or by soaking with ligands (details in Table 2). The crystallization drops were seeded from previously formed crystals of *Mtb*-AnPRT, by streak seeding using a cat whisker. Data collection occurred within a few days after crystallization. In preparation for data collection, crystals were typically soaked in a reservoir solution containing cryoprotectant 15% PEG4000 and appropriate ligands (details in Table 2), before being flash-cooled in liquid nitrogen.

### 2.3 Data collection, structure solution and refinement

Data were collected at the Australian Synchrotron, Beamlines MX1 and MX2 at 110 K. The crystals of the *Mtb*-AnPRT complexes diffracted to a maximum resolution that varied between 1.75 and 2.15 Å. X-ray diffraction spots were indexed and integrated with XDS [14] and scaled with AIMLESS [11]. The high resolution cut-off was determined based on a correlation coefficient (CC_1/2_) [16] exceeding 0.5, with mean *I/σ* between 1 and 1.5, and a *R*_p.i.m._ of 0.7 or less, as calculated by AIMLESS [11]. *R*_p.i.m._ is defined as ∑*hkl* [1/(n−1)]×*R*_merge_, with *R*_merge_ defined as ∑*hkl*|*I*(*hkl*)-<*I*(*hkl*)>|/∑*hkl*<*I*(*hkl*)>, where <*I*(*hkl*)> is the mean of symmetry-equivalent reflections of *I*(*hkl*) [17]. Data and refinement statistics are given in Table 5, Table 6.

*Mtb*-AnPRT (chain A of 3QR9 [2]), without solvent or ligands, and with loops I and II removed, was used as the search model for structure determination by molecular replacement using Phaser [18]. Refinement and model building was performed with COOT [19], Refmac5 [20], and PHENIX [21]. After positioning waters, omit maps were examined (Fig. 2) and ligands placed, with subsequent refinement including restraints for the ligands generated by phenix.elbow [21]. Restraints on protein bond lengths and angles were based on the ideal values of Engh and Huber [15] and model quality was assessed using MolProbity [22]. Figures illustrating structural details were prepared using PyMOL.

The *F*_o_-*F*_c_ map calculated (green, contoured at 3 σ) prior to the addition of ligands to the model for A) **8k**, B) **8j** and C) **8i** (PDB entries 5BNE, 5BO3 and 5BO2, respectively). The 2*F*_o_-*F*_c_ map calculated (blue, contoured at 1 σ) after final refinement, with model including the ligands for D) **8k** (black), E) **8j** (green) and F) **8i** (pink).

## References

[1] Evans G.L., Furkert D.P., Abermil N., Kundu P., de Lange K.M., Parker E.J., Brimble M.A., Baker E.N., Lott J. Shaun (2017). Anthranilate phosphoribosyltransferase: Binding determinants for 5'-phospho-alpha-D-ribosyl-1'-pyrophosphate (PRPP) and the implications for inhibitor design. Biochim. Biophys. Acta.

[2] Castell A., Short F.L., Evans G.L., Cookson T.V., Bulloch E.M., Joseph D.D., Lee C.E., Parker E.J., Baker E.N., Lott J.S. (2013). The substrate capture mechanism of *Mycobacterium tuberculosis* anthranilate phosphoribosyltransferase provides a mode for inhibition. Biochemistry.

[3] Wukovitz S.W., Yeates T.O. (1995). Why protein crystals favour some space-groups over others. Nat. Struct. Biol..

[4] Lee C.E., Goodfellow C., Javid-Majd F., Baker E.N., Shaun Lott J. (2006). The crystal structure of TrpD, a metabolic enzyme essential for lung colonization by *Mycobacterium tuberculosis*, in complex with its substrate phosphoribosylpyrophosphate. J. Mol. Biol..

[5] Evans G.L., Gamage S.A., Bulloch E.M., Baker E.N., Denny W.A., Lott J.S. (2014). Repurposing the chemical scaffold of the anti-arthritic drug lobenzarit to target tryptophan biosynthesis in *Mycobacterium tuberculosis*. Chembiochem.

[6] Cookson T.V., Evans G.L., Castell A., Baker E.N., Lott J.S., Parker E.J. (2015). Structures of *Mycobacterium tuberculosis* anthranilate phosphoribosyltransferase variants reveal the conformational changes that facilitate delivery of the substrate to the active site. Biochemistry.

[7] Cookson T.V., Castell A., Bulloch E.M., Evans G.L., Short F.L., Baker E.N., Lott J.S., Parker E.J. (2014). Alternative substrates reveal catalytic cycle and key binding events in the reaction catalysed by anthranilate phosphoribosyltransferase from *Mycobacterium tuberculosis*. Biochem. J..

[8] Marino M., Deuss M., Svergun D.I., Konarev P.V., Sterner R., Mayans O. (2006). Structural and mutational analysis of substrate complexation by anthranilate phosphoribosyltransferase from *Sulfolobus solfataricus*. J. Biol. Chem..

[9] Krissinel E., Henrick K. (2004). Secondary-structure matching (SSM), a new tool for fast protein structure alignment in three dimensions. Acta Crystallogr. D Biol. Crystallogr..

[10] Ortega A., Amoros D., Garcia de la Torre J. (2011). Prediction of hydrodynamic and other solution properties of rigid proteins from atomic- and residue-level models. Biophys. J..

[11] Evans P.R. (2011). An introduction to data reduction: space-group determination, scaling and intensity statistics. Acta Crystallogr. D Biol. Crystallogr..

[12] Lebedev A.A., Isupov M.N. (2014). Space-group and origin ambiguity in macromolecular structures with pseudo-symmetry and its treatment with the program Zanuda. Acta Crystallogr. D Biol. Crystallogr..

[13] Zwart P.H., Grosse-Kunstleve R.W., Lebedev A.A., Murshudov G.N., Adams P.D. (2008). Surprises and pitfalls arising from (pseudo)symmetry. Acta Crystallogr. D Biol. Crystallogr..

[15] Engh R.A., Huber R. (1991). Accurate bond and angle parameters for X-ray protein-structure refinement. Acta Crystallogr. Sect. A.

[14] Kabsch W. (2010). XDS. Acta Crystallogr. D Biol. Crystallogr..

[16] Karplus P.A., Diederichs K. (2012). Linking crystallographic model and data quality. Science.

[17] Weiss M.S. (2001). Global indicators of X-ray data quality. J. Appl. Crystallogr..

[18] McCoy A.J., Grosse-Kunstleve R.W., Adams P.D., Winn M.D., Storoni L.C., Read R.J. (2007). Phaser crystallographic software. J. Appl. Crystallogr..

[19] Emsley P., Lohkamp B., Scott W.G., Cowtan K. (2010). Features and development of Coot. Acta Crystallogr. D Biol. Crystallogr..

[20] Murshudov G.N., Vagin A.A., Dodson E.J. (1997). Refinement of macromolecular structures by the maximum-likelihood method. Acta Crystallogr. D Biol. Crystallogr..

[21] Adams P.D., Afonine P.V., Bunkoczi G., Chen V.B., Davis I.W., Echols N., Headd J.J., Hung L.W., Kapral G.J., Grosse-Kunstleve R.W., McCoy A.J., Moriarty N.W., Oeffner R., Read R.J., Richardson D.C., Richardson J.S., Terwilliger T.C., Zwart P.H. (2010). PHENIX: a comprehensive Python-based system for macromolecular structure solution. Acta Crystallogr. D Biol. Crystallogr..

[22] Chen V.B., Arendall W.B., Headd J.J., Keedy D.A., Immormino R.M., Kapral G.J., Murray L.W., Richardson J.S., Richardson D.C. (2010). MolProbity: all-atom structure validation for macromolecular crystallography. Acta Crystallogr. D Biol. Crystallogr..

