# Supplementary material for: Datasets, processing and refinement details for Mtb-AnPRT: inhibitor structures with various space groups
Source: Data Brief. 2017 Oct 31;15:1019–29. doi: 10.1016/j.dib.2017.10.051 (PMC5686470; doi:10.1016/j.dib.2017.10.051)
Supplement: Supplementary file 1 — Supplementary material [file mmc1.pdf]

CONFLICT OF INTEREST STATEMENT

We wish to confirm that there are no known conflicts of interest associated with the publication entitled "Datasets, processing and refinement details for *Mtb*-AnPRT: inhibitor structures with various space groups" and there has been no financial support for this work that could have influenced its outcome.

The Corresponding Authors, Dr. Genevieve Evans and Assoc Prof Shaun Lott, are the sole contacts for the Editorial process (including Editorial Manager and direct communications with the office). They are responsible for communicating with the other authors about progress, submissions of revisions and final approval of proofs.

We confirm that we have provided a current, correct email addresses that are accessible to the Corresponding Authors:
g.evans@auckland.ac.nz
s.lott@auckland.ac.nz
